# Diagnostics of Thyroid Malignancy and Indications for Surgery in the Elderly and Younger Counterparts: Comparison of 3,749 Patients

**DOI:** 10.1155/2017/1012451

**Published:** 2017-10-16

**Authors:** Krzysztof Kaliszewski, Dorota Diakowska, Marta Strutyńska-Karpińska, Beata Wojtczak, Michał Aporowicz, Zdzisław Forkasiewicz, Waldemar Balcerzak, Tadeusz Łukieńczuk, Paweł Domosławski

**Affiliations:** ^1^1st Department and Clinic of General, Gastroenterological and Endocrine Surgery, Wroclaw Medical University, Wroclaw, Poland; ^2^Department of Nervous System Diseases, Faculty of Health Science, Wroclaw Medical University, Wroclaw, Poland; ^3^Department and Clinic of Gastrointestinal and General Surgery, Wroclaw Medical University, Wroclaw, Poland

## Abstract

**Background:**

It seems valuable for clinicians to know if diagnostics of thyroid malignancy (TM) and indications for surgery in the elderly patients differ from these in younger counterparts.

**Materials and Methods:**

Retrospective analysis of the medical records of 3,749 patients surgically treated for thyroid tumor. Data of patients with histopathology confirmed TM (*n* = 309) were studied.

**Results:**

The rate of cytological prediction to malignancy was more than three times higher in elderly women. Compression was a main reason for surgery in the elderly (*p* < 0.0001). The final diagnosis of malignancy was significantly higher in older women (*p* = 0.002). Clinical suspicion of malignancy was positively correlated with histopathological diagnosis in total group of women (*r* = 0.543, *p* < 0.001) and total group of men (*r* = 0.560, *p* < 0.001). The subgroup of the eldest TM patients included a significantly higher number of subjects with advanced cancer and primary tumor progression (*p* < 0.0001). Distant metastases were significantly more presented among the elderly patients (*p* = 0.032).

**Conclusions:**

The rate of cytological prediction to malignancy in elderly women is high. Tracheal compression is a common surgical indication in the elderly patients. The final diagnoses of malignancy predominate in elderly women. The oldest TM patients present a higher number of advanced thyroid tumors and distant metastases.

## 1. Introduction

The proportion of the elderly people to the younger population has increased by 90% over the last 30 years, and as some authors predict, by the year 2020, the proportion of the people over 65 years will increase from 12.4% to 20% [1]. According to the same source of information, the number of older United States citizens will reach 80 million by 2050. The other authors estimated that if in 2000 in the world there were 600 million people in age of 60 years or more, in 2050 there will be 2 billion [2]. The United State Census Bureau announced that the number of the elderly Americans aged above 65 years increases by 2.8% per year between 2010 and 2030 [1]. What is more, it is estimated that by 2050 the population above 85 years old will comprise 24% of the elderly in the United States and 5% of the total population in the world [3]. Some authors noticed that approximately 50% of all surgical interventions are performed in patients in age of 65 or older [4].

Benign and malignant thyroid nodules occur with increasing frequency in the elderly patients [5, 6]. Mazzaferri [7] assessed that, by the age of 65, about 50% of these patients have ultrasound revealed nodules, and the same observations are described on basis autopsies performed for the general population [8]. Some other studies say that about 90% of women after the age of 60 years demonstrate the thyroid nodules and about 60% of men after the age of 80 [2]. It was estimated that about 5% of palpable nodules appear malignant on histopathological examination; however some authors revealed strong association between age and malignant potential of thyroid nodules [9]. From 1973, we have observed the rapid increase of incidence of thyroid malignancy in older patients, and it is due to rapid increase of papillary thyroid carcinoma incidence [10]. Besides the fact that this type of cancer has excellent prognosis in general population, a lot of studies suggest that well-differentiated thyroid carcinomas comprised of papillary, follicular, and Hurtle cell carcinomas are more aggressive tumors in elderly patients and are more likely to recur. Sorrenti et al. [11] also confirmed that well-differentiated thyroid carcinoma patients have a favourable prognosis, but they added too that the aggressive thyroid tumors are more frequently observed in the geriatric ages showing a higher disease specific mortality. In older patients, sporadic medullary thyroid carcinomas are more characteristic than hereditary tumors and older age is assessed as poor prognostic factor [12]. Lerch et al. [13] noticed that the age of diagnosis of thyroid carcinoma influences its prognosis. Anaplastic thyroid carcinoma tends to have an extremely poor prognosis in elderly people and most often is observed in the 6th-7th decades of life [14]. Sautter-Bihl et al. [15] revealed that none of the patients in age below 40 years died, while in the group of patients over 60 years old a ten-year survival rate was 79%.

Diagnostics and treatment of thyroid carcinoma is based on the similar surgical procedures in elderly patients as in younger ones. A lot of studies revealed that the most common indications for surgery in older patients are similar than in younger counterparts and the most common are thyroid carcinoma suspicion, multinodular goiter (MNG) with trachea compression, or hyperthyroidism [16]. However, to establish the accurate diagnosis in the elderly patients may seem much more difficult. The clinical dilemmas concern the elderly patients with thyroid malignancy in the early stage of disease. Some authors suggest that older patients generally have a much “stronger” or more urgent indications for surgery like severe compressive or trachea infiltration symptoms due to thyroid carcinoma [17]. The proper diagnosis and accurate indication for surgery in older patients in aspect of some recent performed clinical analyses seem extremely valuable, especially that, as the authors say, thyroid surgery in older patients is associated with higher risk of severe complications than in younger ones [18].

All these observations forced the authors of this study to estimate how more difficult and complicated is to establish the proper diagnosis of thyroid pathology in elderly patients and, next if needed, properly qualify to surgery. The authors analyzed the most common thyroid pathology and the most common indication for surgery in the elderly patients.

## 2. Materials and Methods

Our study protocol was approved by the Bioethics Committee of Wroclaw Medical University (signature number: KB-487/2017).

### 2.1. Demographic and Clinical Characteristics

The terms of “geriatric” or “elderly” patients are not clearly defined. The cutoff age for this group of patients may vary from 60 to 80 years or even older [18]. In our study, we defined the elderly patients as 65 and older.

### 2.2. Indication for Surgery

In aspect of some studies [19], which have revealed the higher risk of perioperative complications among older patients undergoing thyroid surgery, the indications for strumectomy in our patients were accurately analyzed and based on the classical standards.

### 2.3. Study Group

Study group consisted of 3,749 patients with solitary and multiple thyroid nodules, who were surgically treated between January 2008 and December 2015 at Department of General, Gastroenterological and Endocrine Surgery of Wroclaw Medical University, Poland. The average age of the patients was 51 ± 14 years, and female to male ratio was 3,148/601. All patients before the surgical ward admission were diagnosed clinically by ultrasonography (US), computed tomography (CT), or magnetic resonance (MR). All of the patients had ultrasound guided fine needle aspiration biopsy (UG-FNAB) of the thyroid tumor performed minimum once and one month before admission. Clinical and pathological c/pTNM staging was estimated according to AJCC/UICC 2010 7th Edition. The characteristic of the patients in the study group is demonstrated in Table 1.

Total study group of patients (*n* = 3,749) was divided into three subgroups according to the age parameter. The first subgroup combined the patients below 45 years old (“<45”), the second one combined the patients at the age equal to or above 45 years to below 65 years (“≥45–<65”), and the third subgroup combined the patients at the age equal to or above 65 years (“≥65”). Since the first statistical analysis showed that these three subgroups of patients are significantly different in respect of gender parameter (*p* = 0.019), next comparative statistics were performed separately in female and male.

In the second part of the research, selected from among 3,749 subjects, data of patients with histopathology confirmed TM (*n* = 309) were studied. This group was also divided into three subgroups according to the age parameter (the classification was identical to the one performed in the first part of the study). There were no significant differences in the gender distribution between subgroups of TM patients (*p* = 0.520). Therefore receiver operating characteristic (ROC) and comparative analyses of the demographic, clinical, and histopathological parameters were performed for these three subgroups.

### 2.4. Statistical Analysis

Numbers and percentages were calculated for qualitative variables, and averages and standard deviations were measured for quantitative variables. The data distribution was tested using chi-square test. Frequencies were analyzed using Fisher exact test or chi-square test with Yates correction. Correlation analysis was conducted with Spearman test.

The diagnostic potential of clinical examinations in thyroid cancer occurrence in each subgroup was determined by ROC analysis. Results were expressed as area under ROC curve (AUC). The sensitivity, specificity, accuracy, positive predictive value (PPV), negative predictive value (NPV), likelihood ratio of positive results (LR(+)), likelihood ratio of negative results (LR(−)), and Youden's index were also calculated.

In a two-tailed test, significance level *α* ≤ 0.05 was considered as statistically significant. Statistical analyses were performed using STATISTICA 12.0 software (Statistica, Tulsa, OK, USA) and MedCalc 16.8 software (MedCalc Statistical Software, Ostend, Belgium).

## 3. Results

### 3.1. Demographic, Clinical, and Pathological Characteristics of Patients with Thyroid Nodules

We observed significant differences in gender parameter between the three subgroups (female/male ratio in “<45” group: 85/15, in “≥45–<65” group: 85/15, and in “≥65” group: 80/20; *p* = 0.019), so we analyzed data for women and men separately (Table 2).

The rate of cytological prediction to malignancy was more than three times higher in elderly women compared with women below 65 years old. Also follicular neoplasm samples were observed more frequently in women, who were over 65 years old. As it has been shown in comparative analysis of clinical examinations, older women had significantly higher prediction and suspicion to TM than women below 65 years (for both *p* < 0.0001). These results were not observed in subgroup of older men (for both *p* > 0.05) (Table 2).

Goiter enlargement and cosmetics indication were significant factors for operation in women and men below 45 years old, and compression was a main reason for surgery in more than 85% of older women and men. These differences were statistically significant between age subgroups in women and men (for both *p* < 0.0001) (Table 2).

The histopathological data demonstrated that significantly more patients with age below 65 years had extreme results of diagnosis: simple goiter or adenoma (*p* < 0.0001 and *p* < 0.001, respectively, for women and men), and percent value of final diagnosis of malignancy was significantly higher in older women than in women below 65 years old (*p* = 0.002). There were no significant differences in final diagnosis between age subgroups in men (Table 2).

Clinical suspicion of malignancy was positively correlated with histopathological diagnosis not only in total group of women (*r* = 0.543, *p* < 0.001) and total group of men (*r* = 0.560, *p* < 0.001), but also in each of age subgroups (Table 3).

### 3.2. Diagnostic Potential of Clinical Examinations in Prediction of TM Presence in Patients with Thyroid Nodules

The diagnostic potential of clinical examinations in TM occurrence was evaluated in terms of the capacity to rule out malignancy in patients with benign thyroid disease (the controls). The overall accuracy of clinical examinations in prediction of TM presence was high in all subgroups of patients (Table 4).

### 3.3. Demographic, Clinical, and Pathological Characteristics of Patients with TM

As shown in Table 5, the subgroup of the eldest TM patients included a significantly higher number of subjects with advanced cancer (III or IV stage of disease progression) and with primary tumor progression (T3 and T4) (for both *p* < 0.0001). Distant metastases were significantly more presented among the elderly patients in comparison to patients below 65 years old (*p* = 0.032). No significant differences were observed in type of surgery, necessity of reoperation, type of nodule, and lymph node metastases between the three subgroups of TM patients (Table 5).

## 4. Discussion 

The prevalence of thyroid malignancy increases with age and in the elderly it is more aggressive process [20, 21]. It was estimated that papillary and follicular thyroid carcinomas are more aggressive tumors after 45 years and anaplastic thyroid carcinoma is extremely rare observed before the age of 60 [22]. Some authors revealed that, in patients in age of 65 and older, more aggressive tumors with extrathyroid spread, multiple, larger lesion, and more advanced-stage disease can be observed [23]. They added that nonpapillary types of carcinoma were most often observed.

Diagnostics and qualification for thyroid surgery of elderly patients still remain a set of challenges. The effect of age on the risk of thyroid surgery still remains under debate. In the literature, there are opposite opinions. Some authors suggest that there is no higher risk of complications in thyroid surgery for elderly patients [2], whereas the others say that the possibility for surgical complication is very high [21]. Some authors suggest that thyroid surgery presents various risks for older people [24]. On the basis of all these opinions we think that proper diagnostics and qualification to surgery in the elderly patients are extremely important. Majority of authors agree that there are two main indications for thyroid surgery in elderly patients. The first is mechanical compression symptoms caused by solitary tumor or goiter, often localized in retrosternal space. The second is suspicion or verified malignant process [21, 25–27]. The indications for thyroid surgery like compressive syndrome, thyrotoxicosis, and recent gland enlargement were described as more common in elderly patients than in younger population (the percentage in age above 70 years was 38.2%, 30.9%, and 27.3%, respectively) [26]. The basic diagnostic tool for thyroid tumors to rule out or confirm its malignancy still remains UG-FNAB. A large number of studies have demonstrated the high overall accuracy of UG-FNAB for evaluation of thyroid nodules, and this finding has been confirmed, particularly for patients with solitary nodules [28]. Some authors revealed that older patients underwent thyroid surgery more often due to suspicion or verified malignancy than younger patients (52.7% versus 30.3%) [27]. The next more common indication is compression of trachea (38.2% versus 3.1%). The same authors say that benign, asymptomatic MNG in opposite to younger population is very rare indication for strumectomy in elderly patients (9.1% versus 66.6%). However in the literature there are no clear data about differences of extent of surgery between each age group of patients. In a very interesting study performed by Ríos et al. [19], they analyzed 591 patients with 81 individuals above 65 years old, who underwent thyroidectomy due to MNG. They revealed that elderly patients more often than younger ones presented compressive symptoms (43% versus 21%) and rare suspicion for malignancy (19% versus 29%), recent goiter growth (1% versus 6%), or patient request (4% versus 12%). In our study the main indication for surgery in the elderly patients group was compression symptom. The second was verified malignant tumor or suspicion of malignancy. The number of retrosternal goiters in elderly patients was significantly higher than in younger ones. Only in this group of patients we observed urgent indication for surgery, which had to be performed immediately after admission to our department. Park et al. [29] performed very interesting study, in which they compared three age groups of patients who received surgery due to well-differentiated thyroid carcinoma. They analyzed patients between 45 and 64, 65 and 79, and 80 years and older. They noticed that patients in the age of 65 years and older had more aggressive malignant disease with multiple, larger tumors and more advanced-stage disease. In this group of patients, in opposite to younger ones, there were a lot of nonpapillary thyroid carcinoma and extraglandular extension. The same authors noticed that these elderly patients underwent less radical treatment without radioiodine ablation therapy even if American Thyroid Association guidelines recommended more aggressive treatment [30]. In a very similar observation described by Panigrahi et al. [31], they analyzed 2,033 patients surgically treated due to medullary thyroid carcinoma. Among all patients without local invasion and distant metastases, in the group of patients above 65 years, the authors noticed the fewest percentage of individuals receiving recommended treatment. In the group of patients below 40 years old it was 65% compared to those above 65, where it was only 45%.

In the other study, the authors analyzed the most common indications for thyroid surgery in the three age groups of patients: 50–60 years (725 patients), 61–74 years (685 patients), and more than 75 years (221 patients). They noticed that the most common indication in all groups was retrosternal goiter with tracheal compression, but it was least frequent in the oldest group. In this group there were the most of remedial surgeries [25]. Raffaelli et al. [32] analyzed the indications for thyroid surgery in 320 patients in the age of 70 years and more. They noticed that the most common indication was bilateral multinodular goiter, next suspicion or confirmed malignant process, and toxic goiter. In similar observations revealed by Lang and Lo [26], they confirmed that in patients aged 70 years and more the most common indication for thyroid surgery was retrosternal goiter, but they added that in this group of patients the volume of goiter was significantly higher.

The next very important aspect of thyroid surgery in elderly patients is emergency thyroidectomies. This surgical procedure is indicated in case of severe respiratory distress caused by airway compression. Miccoli et al. [33] revealed that the most common reasons of acute airway compression in patients above 80 years were malignant process. The presence of retrosternal goiter or its mediastinal location very often causes the trachea compression with respiratory failure. In our clinic during the analysis period we treated 11 patients with the huge retrosternal goiter which caused acute compression symptoms. However retrosternal goiter not always causes trachea compression. In the asymptomatic patients, especially in the geriatric age, decision for surgery is extremely difficult. In aspect of some recent studies, which suggest that in the elderly patients the risk of complication after thyroid surgery is high, it is very valuable to identify the patients who become symptomatic without surgery, those who benefit from only observation, and in the end those who need rapid surgical procedure. In our study we operated on 5 patients requiring emergency admission and emergency surgery due to acute airway obstruction from a large compressive goiter.

The next important issue of indications and thyroid surgery in the elderly patients is secondary operations. These might be performed mainly due to two basic clinical situations. The first is local or lymph node recurrence of thyroid malignant process and the second is recurrent goiter with compression symptoms. Some authors say that the number of secondary thyroid operations is significantly higher in elderly patients than in younger group [25, 27]. These observations were confirmed in our study.

In the previous analyses, it can be observed that some authors pay attention to a very important aspect of the older people living, that is, a quality of life [34]. This problem seems extremely important especially in elderly patients, where surgery not always increases the quality of life. In our study, we treated 6 patients where the only surgical procedure that we could perform was tracheostomy and gastrostomy. All individuals had advanced malignant processes qualified as inoperable cases. Matsuyama et al. [34] performed 85 surgical procedures in elderly patients with thyroid carcinoma and revealed that successful operation in these patients improved the quality of life.

Mekel et al. [21] analyzed indications for thyroid surgery of 332 patients. They divided the patients as octogenarian and younger patients. The authors did not reveal any differences in preoperative indications for surgery. In both groups there were equal numbers of indications like benign diseases, suspected malignancy, and follicular neoplasms.

The next issue of thyroid surgery in elderly patients is the higher prevalence of more aggressive types of thyroid carcinoma than in younger patients. Dellal et al. [20] described their observations performed in 933 patients. They noticed that the rates of anaplastic and Hurtle cell cancers were increased in geriatric ages. These authors concluded that cytological evaluation of thyroid lesions should be especially considered because of increased tendency for aggressive thyroid cancer types in elderly patients. We confirmed these observations in our study. We noticed significantly higher number of individuals with aggressive thyroid malignant tumors in the subgroup of patients in age of 65 and older.

## 5. Conclusions

The rate of cytological prediction of malignancy in elderly women is high. Tracheal compression is a common surgical indication in the elderly patients. The final diagnoses of malignancy predominate in elderly women, but not in men. The oldest thyroid cancer patients present a higher number of advanced thyroid tumors and distant metastases, but not lymph node metastases. Undifferentiated carcinomas, sarcomas, secondary tumors, and lymphomas of thyroid occur with increasing frequency in comparison to younger counterparts; however well-differentiated carcinomas predominate. Retrosternal goiter is a common surgical indication in the elderly patients. Every thyroid pathology in elderly patients should be taken under careful consideration as the prevalence of malignant tumors with more aggressive course.

## Figures and Tables

**Table 1:** Baseline characteristics of 3,749 patients with thyroid nodules.

Parameters	Mean ± SD or *n* (%)
Age (years)	51.4 ± 14.4
*Gender*	
Female	3,148 (84.0)
Male	601 (16.0)
*Cytological diagnosis according to TBSRTC*	
Stage II (normotype thyrocytes, lymphocytes, thyroiditis suspicion)	3,206 (85.5)
Stage III (AUS/FLUS)	84 (2.2)
Stage IV (follicular neoplasm)	225 (6.0)
Stage V (malignancy suspicion)	48 (1.3)
Stage V (malignancy/lymphoma suspicion)	2 (0.1)
Stage VI (papillary carcinoma)	178 (4.7)
Stage VI (medullary carcinoma)	6 (0.2)
*Clinical suspicion of malignancy*	
No	3,290 (87.8)
Yes	459 (12.2)
*Histological diagnosis*	
Benign multinodular goiter	2,946 (78.6)
Thyroiditis	118 (3.1)
Follicular carcinoma	25 (0.7)
Papillary carcinoma	247 (6.6)
Medullary carcinoma	10 (0.3)
Undifferentiated thyroid carcinoma	9 (0.3)
Secondary malignant tumor	4 (0.1)
Lymphoma	9 (0.2)
Follicular adenoma	375 (10.0)
Squamous cell carcinoma	1 (0.03)
Abscess	1 (0.03)
*Final diagnosis*	
Benign	3,440 (91.8)
Malignant	309 (8.2)

TBSRTC: The Bethesda System for Reporting Thyroid Cytology, Second Edition 2010 Bethesda, Maryland; AUS: atypia of undetermined significance; FLUS: follicular lesion of undetermined significance.

**Table 2:** Distribution of cases according to gender and age parameters. Data were presented as number (percent).

	Female				Male			
	<45 years (*n* = 995)	≥45–<65 years (*n* = 1584)	≥65 years (*n* = 569)	*p* value	<45 years (*n* = 178)	≥45–<65 years (*n* = 285)	≥65 years (*n* = 138)	*p* value
*Cytological diagnosis according to TBSRTC*								
Stage II (normotype thyrocytes, lymphocytes, thyroiditis suspicion)	841 (84.5)	1,382 (87.3)	448 (78.7)	<0.0001^**∗**^	151 (84.8)	261 (91.6)	123 (89.1)	0.254
Stage III (AUS/FLUS)	26 (2.6)	34 (2.2)	13 (2.3)		5 (2.8)	4 (1.4)	2 (1.5)	
Stage IV (follicular neoplasm)	65 (6.5)	87 (5.5)	50 (8.8)		12 (6.7)	8 (2.8)	3 (2.2)	
Stage V (malignancy suspicion)	3 (0.3)	15 (1.0)	21 (3.7)		3 (1.7)	2 (0.7)	4 (2.9)	
Stage V (malignancy/lymphoma suspicion)	0 (0.0)	0 (0.0)	2 (0.4)		0 (0.0)	0 (0.0)	0 (0.0)	
Stage VI (papillary carcinoma)	59 (5.9)	62 (3.9)	34 (6.0)		7 (3.9)	10 (3.5)	6 (4.4)	
Stage VI (medullary carcinoma)	1 (0.1)	4 (0.3)	1 (0.2)		0 (0.0)	0 (0.0)	0 (0.0)	

*Clinical suspicion of malignancy*								
No	867 (87.1)	1,416 (89.4)	461 (81.0)	<0.0001^**∗**^	156 (87.6)	265 (93.0)	125 (90.6)	0.157
Yes	128 (12.9)	168 (10.6)	108 (19.0)		22 (12.4)	20 (7.0)	13 (9.4)	

*Indication for surgery (out of malignancy) *								
Trachea compression	278 (32.1)	720 (50.9)	444 (96.3)	<0.0001^**∗**^	46 (30.0)	117 (43.8)	112 (89.6)	<0.0001^**∗**^
Recent tumor/goiter enlargement	205 (23.6)	284 (20)	8 (1.7)		79 (50.0)	49 (18.8)	7 (5.6)	
Urgent indication (acute respiratory failure/retrosternal goiter)	0 (0.0)	0 (0.0)	7 (1.5)		0 (0.0)	0 (0.0)	4 (3.2)	
Cosmetic indication	384 (44.3)	412 (29.1)	2 (0.4)		31 (20.0)	99 (37.5)	2 (1.6)	

*Histological follow-up *								
Benign multinodular goiter	759 (76.3)	1,281 (81.0)	433 (76.1)	<0.0001^**∗**^	131 (73.6)	236 (82.8)	105 (76.1)	0.001^**∗**^
Thyroiditis	36 (3.6)	50 (3.2)	20 (3.5)		7 (3.9)	3 (1.1)	2 (1.5)	
Follicular carcinoma	7 (0.7)	5 (0.3)	5 (0.9)		0 (0.0)	1 (0.4)	7 (5.1)	
Papillary carcinoma	76 (7.6)	96 (6.1)	44 (7.7)		9 (5.1)	18 (6.3)	4 (2.9)	
Medullary carcinoma	2 (0.2)	2 (0.1)	3 (0.5)		2 (1.1)	1 (0.4)	0 (0.0)	
Undifferentiated thyroid cancer	0 (0.0)	1 (0.1)	6 (1.1)		0 (0.0)	1 (0.4)	1 (0.7)	
Secondary malignant tumor	0 (0.0)	1 (0.1)	2 (0.4)		0 (0.0)	0 (0.0)	1 (0.7)	
Lymphoma	0 (0.0)	2 (0.1)	6 (1.1)		0 (0.0)	1 (0.4)	0 (0.0)	
Squamous cell carcinoma	0 (0.0)	0 (0.0)	1 (0.2)		0 (0.0)	0 (0.0)	0 (0.0)	
Adenoma	115 (11.6)	142 (9.0)	47 (8.3)		29 (16.3)	24 (8.4)	18 (13.0)	
Abscess	0 (0.0)	1 (0.1)	0 (0.0)		0 (0.0)	0 (0.0)	0 (0.0)	

*Final diagnosis*								
Benign	910 (91.5)	1,474 (93.1)	501 (88.1)	0.002^**∗**^	167 (93.8)	263 (92.3)	125 (90.6)	0.561
Malignant	85 (8.5)	110 (6.9)	68 (11.9)		11 (6.2)	22 (7.7)	13 (9.4)	

*Recurrent goiter*								
No	995 (100.0)	1,572 (99.2)	472 (83.0)	<0.0001^**∗**^	178 (100.0)	285 (100.0)	130 (94.2)	<0.0001^**∗**^
Yes	0 (0.0)	12 (0.8)	97 (17.0)		0 (0.0)	0 (0.0)	8 (5.8)	

TBSRTC: The Bethesda System for Reporting Thyroid Cytology, Second Edition 2010 Bethesda, Maryland; AUS: atypia of undetermined significance; FLUS: follicular lesion of undetermined significance; ^*∗*^statistically significant.

**Table 3:** Analyses of correlation between clinical suspicion of malignancy and final diagnosis of thyroid malignancy presence.

	Total (*n* = 3,148)		<45 years (*n* = 995)		≥45–<65 years (*n* = 1,584)		≥65 years (*n* = 569)	
	*r*	*p* value	*r*	*p* value	*r*	*p* value	*r*	*p* value
	Female							
Clinical suspicion of malignancy & final diagnosis	0.543	<0.0001^**∗**^	0.581	<0.0001^**∗**^	0.503	<0.0001^**∗**^	0.554	<0.0001^**∗**^

	Male							
Clinical suspicion of malignancy & final diagnosis	0.560	<0.0001^**∗**^	0.542	<0.0001^**∗**^	0.590	<0.0001^**∗**^	0.575	<0.0001^**∗**^

^*∗*^Statistically significant.

**Table 4:** Diagnostic potential of *clinical suspicion of malignancy* parameter in prediction of thyroid malignant tumor presence in total study group and age subgroups of patients with thyroid nodules (ROC analysis).

	Total study group (*n* = 3,749)	<45 years (*n* = 1,173)	≥45–<65 years (*n* = 1,869)	≥65 years (*n* = 707)
AUC	0.825	0.850	0.800	0.829
95% CI	0.790–0.855	0.800–0.900	0.751–0.850	0.772–0.885
*p* value	<0.0001^**∗**^	<0.0001^**∗**^	<0.0001^**∗**^	<0.0001^**∗**^
SE	0.015	0.026	0.025	0.029
Sensitivity	0.72	0.77	0.66	0.75
Specificity	0.93	0.93	0.94	0.90
Accuracy	0.91	0.92	0.92	0.89
LR(+)	10.43	10.92	11.33	7.86
LR(−)	0.30	0.25	0.36	0.27
PPV	0.48	0.49	0.46	0.50
NPV	0.97	0.98	0.97	0.97
Youden's index	0.65	0.70	0.60	0.66

AUC: area under ROC curve; 95% CI: confidence interval; SE: standard error; LR(+): likelihood ratio of positive results; LR(−): likelihood ratio of negative results; PPV: positive predictive value; NPV: negative predictive value; ^*∗*^statistically significant.

**Table 5:** Clinical and pathological characteristics of thyroid malignancy (TM) patients (*n* = 309) divided into three subgroups according to age parameter. Data were presented as *n* (%).

	*N*	<45 years (*n* = 96)	≥45–<65 years (*n* = 132)	≥65 years (*n* = 81)	*p* value
Gender					
Female	263	85 (88,5)	110 (83.3)	68 (84.0)	0.520
Male	46	11 (11.5)	22 (16.7)	13 (16.0)	

Type of surgery					
Radical	202	65 (67.7)	82 (66.7)	46 (63,0)	0.803
Nonradical	107	31 (32.3)	41 (33.3)	27 (37.0)	

Reoperation					
No	217	70 (72.9)	87 (70.7)	50 (70.4)	0.919
Yes	92	26 (27.1)	36 (29.3)	21 (29.6)	

pTNM					
I	199	88 (91.7)	84 (67.2)	23 (31.9)	<0.0001^**∗**^
II	48	5 (5.2)	21 (16.8)	18 (25.0)	
III	31	1 (1.0)	11 (8.8)	15 (20.8)	
IV	31	2 (2.1)	9 (7.2)	16 (22.2)	

pT					
T1a	125	45 (47.9)	62 (49.2)	16 (22.2)	<0.0001^**∗**^
T1b	68	31 (32.9)	26 (20.6)	8 (11.1)	
T2	56	15 (15.9)	19 (15.1)	19 (26.3)	
T3	25	1 (1.0)	8 (6.4)	13 (18.1)	
T4a	17	2 (2.1)	8 (6.4)	4 (5.6)	
T4b	18	0 (0.0)	3 (2.3)	12 (16.7)	

pN					
N0	148	49 (51.0)	60 (47.6)	35 (48.6)	0.071
N1a	42	12 (12.5)	12 (9.5)	14 (19.4)	
N1b	20	5 (5.2)	4 (3.2)	7 (9.7)	
Nx	99	30 (31.3)	50 (39.7)	16 (22.2)	

pM					
M0	218	77 (80.2)	87 (69.1)	54 (75.0)	0.032^**∗**^
M1	6	0 (0.0)	2 (1.6)	4 (5.6)	
Mx	85	19 (19.8)	37 (29.3)	14 (19.4)	

Type of nodule					
Solitary	223	71 (73.9)	88 (69.8)	56 (77.8)	0.468
Multiple	86	25 (26.1)	38 (30.2)	16 (22.2)	

^*∗*^Statistically significant.
